# Porous Graphitic Carbons Containing Nitrogen by Structuration of Chitosan with Pluronic P123

**DOI:** 10.1021/acsami.0c19463

**Published:** 2021-03-11

**Authors:** Lu Peng, Yong Peng, Ana Primo, Hermenegildo García

**Affiliations:** Instituto Universitario de Tecnología Química, Universitat Politècnica de València-Consejo Superior de Investigaciones Científicas, Av. de los Naranjos s/n, 46022 Valencia, Spain

**Keywords:** soft structuration, Pluronic 123, graphitic carbons, chitosan pyrolysis, photocatalytic hydrogen generation

## Abstract

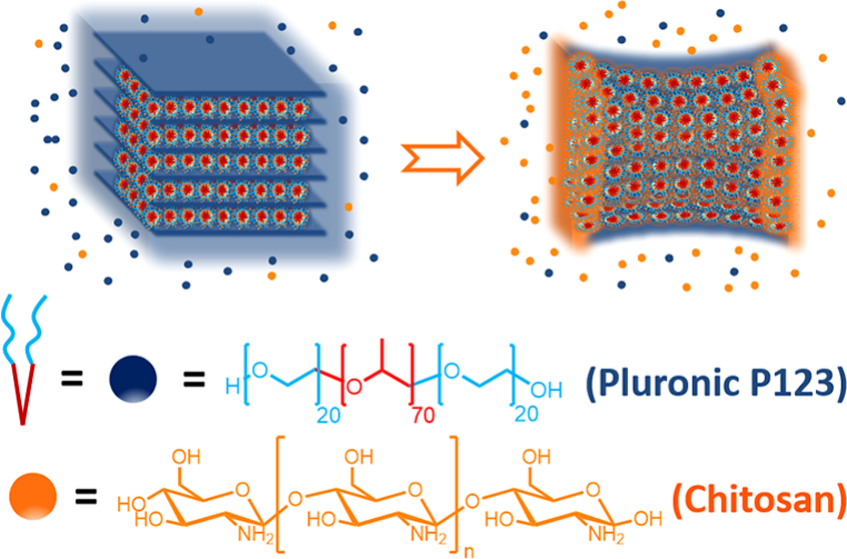

Using Pluronic P123 as a structure-directing agent and chitosan as a carbon precursor, different porous carbons with remarkable morphologies such as orthohedra or spheres with diametrically opposite holes are obtained. These particles of micrometric size are constituted by the stacking of thin sheets (60 nm) that become increasingly bent in the opposite sense, concave in the upper and convex in the bottom hemispheres, as the chitosan proportion increases. TEM images, after dispersion of the particles by sonication, show that besides micrometric graphene sheets, the material is constituted by nanometric onion-like carbons. The morphology and structure of these porous carbons can be explained based on the ability of Pluronic P123 to undergo self-assembly in aqueous solution due to its amphoteric nature and the filmogenic properties of chitosan to coat Pluronic P123 nanoobjects undergoing structuration and becoming transformed into nitrogen-doped graphitic carbons. XPS analysis reveals the presence of nitrogen in their composition. These porous carbons exhibit a significant CO_2_ adsorption capacity of above 3 mmol g^–1^ under 100 kPa at 273 K attributable to their large specific surface area, ultraporosity, and the presence of basic N sites. In addition, the presence of dopant elements in the graphitic carbons opening the gap is responsible for the photocatalytic activity for H_2_ generation in the presence of sacrificial electron donors, reaching a H_2_ production of 63 μmol g^–1^ in 24 h.

## Introduction

It is well known that surfactant, amphiphilic molecules or polymers undergo spontaneous self-assembly forming in an aqueous phase nanoobjects that can be used as soft templates in the preparation of materials with regular pore dimensions.^1−3^ By applying this soft-templating methodology, during the condensation of molecular precursors of inorganic materials, remarkable examples of structuration forming solids with regular porosity have been reported. Thus, soft templates have been widely used in the synthesis of zeolites,^4−7^ mesoporous aluminosilicates,^5,8^ and even single crystals of inorganic metal oxide semiconductors.^9−11^ In this context, the use of soft templates for the preparation of organic materials, although known,^12,13^ is considerably much less documented, probably because of the scarcity of known examples of organic compounds able to coat nanoobjects and the poor solubility of many organic molecules in the aqueous phase in which structuration takes place by hydrophilic/hydrophobic forces. Obviously, there is still room for templation of organic compounds to reach the level of maturity achieved in inorganic materials structuration.

Recently, we have reported that hexadecyltrimethylammonium chloride (CTAC) can be used as a soft template for the preparation of 3D graphitic carbons from sodium alginate, whose walls are constituted by the stacking of a few layers of defective graphene.^14^ Besides CTAC, block copolymers such as polyethylene oxide–polypropylene oxide–polyethylene oxide (Pluronic P123) are also among the most common templating agents in the synthesis of mesoporous silicas,^15,16^ such as SBA-15^17,18^ and related mesoporous materials.^19−22^ Therefore, it appears of interest to expand the soft-templating method of chitosan to obtain graphitic carbons using Pluronic P123 as the soft template agent.

The present study shows that systematic variation of the Pluronic (P) vs chitosan (CS) concentration produces remarkable changes in the morphology of the resulting particles (P@CS) from cubes to spheres derived from the stacking of thin micrometric sheets. This unprecedented control of the morphology of P@CS particles is inherited in the carbon residues obtained by pyrolysis of the cubes and spheres [3D (N)C]. The resulting porous 3D (N)C exhibits a notable CO_2_ adsorption capacity and photocatalytic activity for H_2_ generation.

## Results and Discussion

**Scheme 1:**
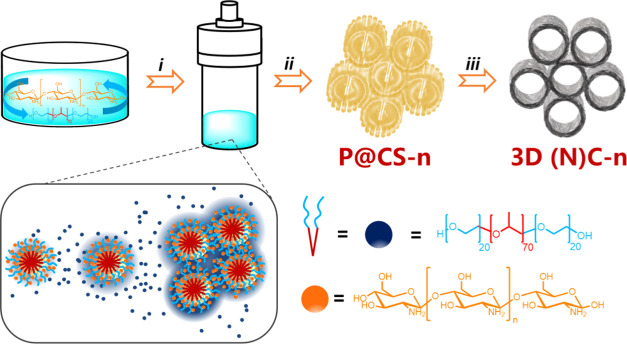
Cartoon Illustrating the Procedure Used for Preparation of P@CS and 3D@(N)C Samples Under Study (i) Hydrothermal treatment of CS dissolved in water containing P, (ii) solvent evaporation, and (iii) pyrolysis under an Ar atmosphere. The drawing shows the structure of P and CS and a cartoon of self-assembly of P into nanoobjects that are overcoated with CS.

Basically, the preparation procedure consists of mixing aqueous solutions of appropriate weights of P as the template and CS, subjecting the mixture to hydrothermal treatment in an autoclave at 100 °C under autogenous pressure for 24 h. After drying, P@CS is transformed into graphitic 3D (N)C carbons by pyrolysis under Ar flow at 900 °C. Scheme 1 illustrates the preparation procedure.

Structuration derived from the combination of two properties, namely, self-assembly of P in water and the ability of CS fibrils to form high-quality films coating curved nanoobjects, even if they are not rigid but as liquid crystals. Formation of the graphitic 3D (N)C carbons results from the well-known transformation of CS into N-doped defective graphene upon hydrothermal treatment at high temperatures.^23^ In this way, it is expected that in the hydrothermal treatment P will form nanoobjects in the aqueous phase due to its spontaneous chain-folding trying to bury the hydrophobic block of the copolymer inside the hydrophilic polyethylene domains. After folding and self-assembly of polyethylene domains in P, filmogenic CS fibrils are expected to cover the surfactant objects due to their ability to form thin films of nanometric thickness and subnanometric roughness (Scheme 1, expansion of hydrothermal treatment).^24^ The last step of the synthesis will consist in the decomposition of P and the transformation of CS into graphitic 3D carbon. Table 1 summarizes the samples prepared in the present study, the corresponding weights of P and CS, and the analytical data of the P@CS or 3D (N)G particles.

**Table 1:** List of Samples Under Study and Their Main Physicochemical Parameters

Sample^a^	*m*_P_ (mg)	*m*_CS_ (mg)	*m*_CS_/*m*_P_ ratio	C (wt %)^b^	N (wt %)^b^	*S* (m^2^ g^–1^)^c^	*Q*_max_ (mmol g^–1^)^d^
P@CS-1	234	325	1.4				
3D (N)C-1				78.66	6.74	477	2.96
P@CS-2	134	400	3.0				
3D (N)C-2				78.02	5.39	499	3.03
P@CS-3	67	450	6.7				
3D (N)C-3				79.11	6.45	402	2.61
P@CS-4	34	475	14.1				
3D (N)C-4				78.13	6.51	441	2.86

a P@CS-*n* and 3D (N)C-*n* refer to the samples before and after pyrolysis, respectively.

b It is assumed that the rest to 100% is residual oxygen.

c Based on CO_2_ adsorption at 273 K.

d *Q*_max_ refers to the CO_2_ adsorption capacity at 273 K and 100 kPa.

**Figure 1:**
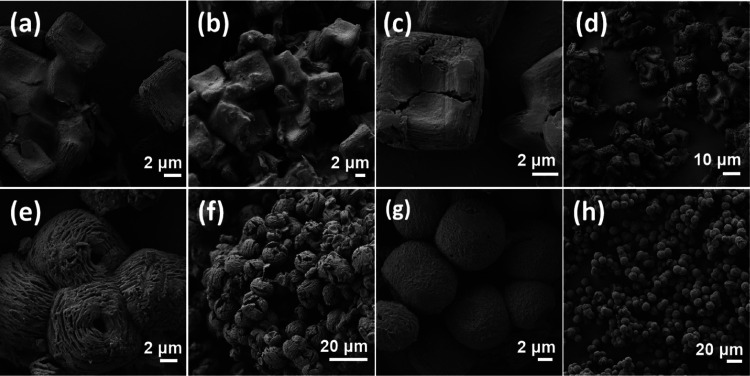
FESEM images of P@CS-*n* corresponding to (a, b) *n* = 1; (c, d) *n* = 2; (e, f) *n* = 3; and (g, h) *n* = 4.

Field-emission scanning electron microscopy (FESEM) images of the P@CS-*n* materials resulting from the self-assembly of P and CS reveal the remarkable difference in the morphology of the particles depending on the mass ratio between P and CS. Figure 1 shows some images corresponding to these samples. For the sample P@CS-1 prepared with the highest P concentration, quasi-orthohedral particles of about 6.4 μm length, 5.8 μm width, and 2.0 μm height average were formed, accompanied by some debris and an overcoat of the excess of the amphiphilic material. Higher magnification of the orthohedral particles shows that they are formed by the stacking of thin sheets of about 60 nm thickness. The upper and bottom surfaces of the orthohedra exhibit some concave/convex curvature, probably revealing the occurrence of some stress forces on the material.

Upon decreasing the amount of P and increasing the mass of CS up to a CS/P mass ratio of 3, the morphology of the orthohedra in P@CS-2 becomes further disturbed, the concave/convex curvature of the upper and bottom faces being more pronounced. Using CS/P proportions of 3 or higher, the excess of the amphiphilic material was much less apparent in the FESEM images, suggesting that P is incorporated within the P@CS-2 material. Importantly, the existence of thin plates as the primary building block of the particles was again observed. Figure 1c,d also includes images of sample P@CS-2.

Upon further increase of the CS mass under exactly the same preparation conditions, the orthohedral shape changes toward quasi-spherical particles of about 14 μm diameter in P@CS-3 and CS-4. These spheres were also constituted by the stacking of two-dimensional plates of 60 nm thickness. Notably, the presence of diametrically opposite holes was also clearly observed in the P@CS-3 and P@CS-4 images presented in Figure 1. Figure S1 in the Supporting Information contains an additional set of images of P@CS.

**Figure 2:**
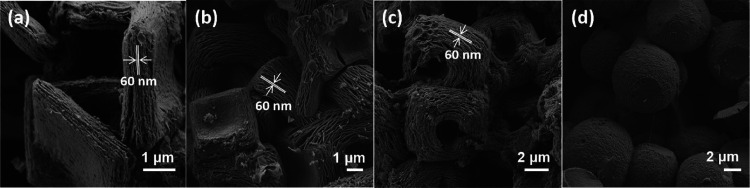
FESEM images of 3D (N)C-*n* corresponding to (a) *n* = 1; (b) *n* = 2; (c) *n* = 3; and (d) *n* = 4.

Following the concept of the present study and in accordance with Scheme 1, samples P@CS-*n* derived from the structuring of CS by P were submitted to pyrolysis under an inert gas at 900 °C. This thermal treatment has been reported in the case of CS films and powders to convert this poly(glucosamine) into N-doped defective graphene.^24^ In previous cases, it has been found that the pyrolysis of CS nanoobjects obtained by employing hard templates also results in the transformation of CS into N-doped graphene maintaining the tridimensional morphology of nanoobjects [3D (N)C].^25^ As it can be seen in Figure 2, the same behavior was observed here with some partial deterioration of the micrometric particles, due to the breakage of small bits of the spongy particle. In the conversion of the P@CS-*n* samples into the corresponding 3D (N)C carbons, some shrinkage in the size of the particles was also observed, again in agreement with the previous reports.^23^ Therefore, in the present case, the samples 3D (N)C-1 and 3D (N)C-2 derived from orthohedral P@CS-1 or -2 particles result in orthohedra of N-doped graphene with a thickness of about 0.8 μm (in comparison to the 2 μm of the precursor). This shrinkage reflects the stacking of the defective graphene sheets in 3D (N)C-1 and -2 samples. Similarly, quasi-spherical particles of P@CS-3 and P@CS-4 obtained at higher CS concentrations become converted into spheres of graphitic carbons 3D (N)C-3 and 3D (N)C-4 of diameter about 10 μm, in comparison of 14 μm for the precursors. Notably, the diametrically opposite holes of the 3D (N)C-3 and 3D (N)C-4 spheres are preserved during the pyrolysis. In the case of the spheres, the diameter of the holes is about 400 nm. Figure S2 in the Supporting Information contains an additional set of FESEM images of 3D (N)C-*n*.

**Scheme 2:**
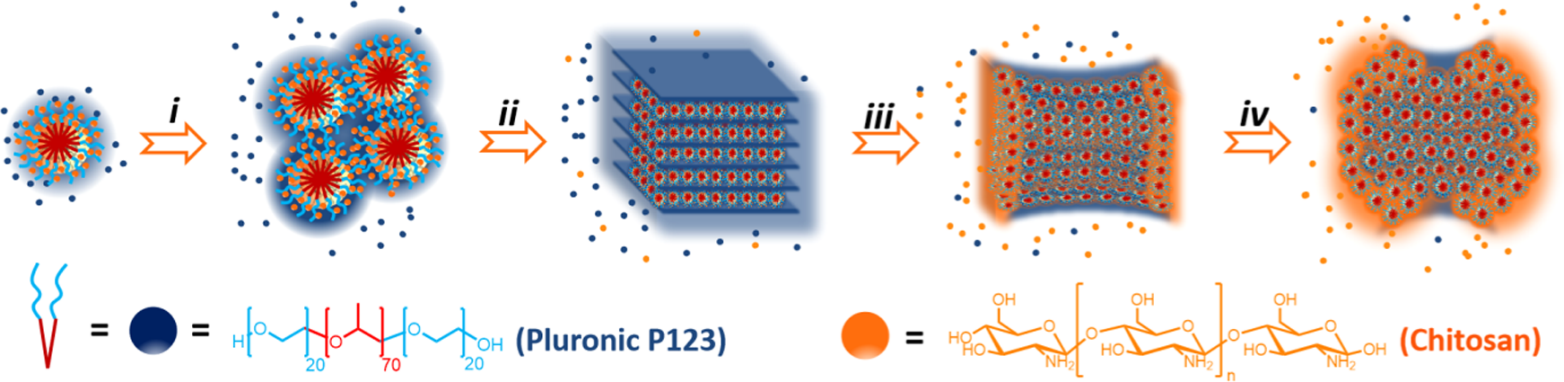
Morphological Changes of 3D (N)C with the Decrease of the P/CS Mass Ratio i) P chain-folding; (ii) formation of lamellar structures; (iii) increasing bending due to the stress caused by CS entering at the layers’ borders; and (iv) morphological change from the square prism to a spherical shape. Pyrolysis of P/CS gives rise to layers and carbon particles at the borders arising from stuffed CS.

A plausible rationalization of the remarkable morphology and morphological changes occurring upon decreasing the P/CS mass ratio is presented in Scheme 2. According to this proposal, the assembly of P and CS tends to form particles constituted by the stacking of layered fibrils of CS assembled on P, the sheets having a natural tendency to form orthohedra. However, the borders of the layers would be subjected to an increasing stress force, the magnitude of these forces growing in the upper and bottom faces. This increasing stress will be responsible for the concave/convex bending of the upper and bottom layers, respectively, as the percentage of CS increases causing their eventual conversion into a hole.

**Figure 3:**
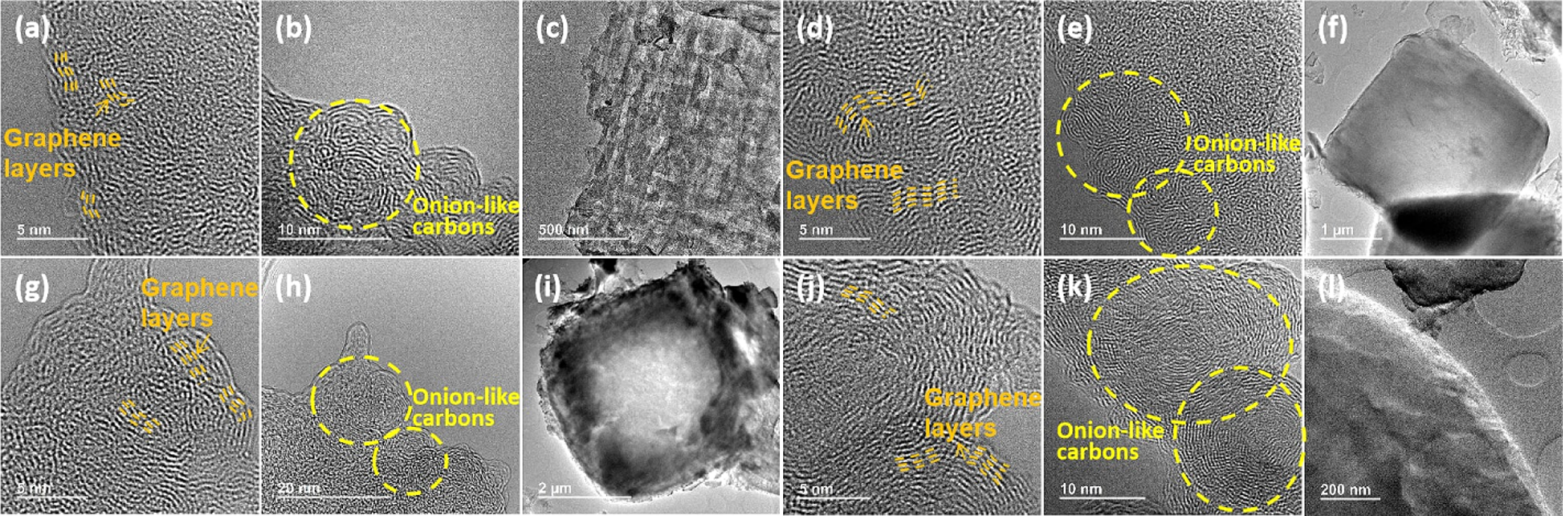
TEM images of 3D (N)C-*n* corresponding to (a–c) *n* = 1; (d–f) *n* = 2; (g–i) *n* = 3; and (j–l) *n* = 4.

To gain further information on the structure and components constituting 3D (N)C-*n*, the carbon residues were submitted to ultrasound treatment and the dispersed fragments were analyzed by transmission electron microscopy (TEM). Sonication produces the destruction of most of the solid carbon residue resulting in the formation of a black ink. The particles present in these inks were imaged by TEM after solvent evaporation. In all cases, images revealed that the carbon residues were composed of two different elements. Figure 3 presents a selection of images to illustrate the two types of particles present in all of the 3D (N)C samples, while Figure S3 in the Supporting Information compiles a more complete set of TEM images for these 3D (N)G carbon residues. It was observed that after sonication, the carbon residues were constituted by large, micrometric sheets, particularly prevalent for samples 3D (N)C-1 and 3D (N)C-2 obtained using larger P amounts and exhibiting in FESEM an orthohedral particle morphology. At higher magnification, the image contrast indicates that the stacking of thinner layers forms these sheets. A closer inspection of these sheets revealed that they are continuous without apparent boundaries or discontinuity. Higher magnification of these sheets allows the observation of the layer stacking expected for N-doped defective graphene reported for CS after pyrolysis.^23^ In addition, the presence of some grains with structural alignment at the nanometric scale was observed on the borders of the sheets.

The second components observed upon ultrasound dispersion of the material are carbon nanoparticles. These nanoparticles correspond to onion-like carbon of about 20 nm average, much smaller than the previously mentioned micrometric sheets. Figure 3 also shows images corresponding to these onion nanoparticles. The distance between the different onion shells was measured as 0.34 nm, corresponding to the interlayer space of graphite in agreement with the graphitic structure of these carbon onions. The presence of these onion-like nanoparticles was observed for the four 3D (N)C carbon samples.

Concerning the origin of these two components, it is proposed that the two elements, micrometric sheets and nanometric onions are derived from CS but not from P. This proposal is based on the thermogravimetric analysis of pure P that shows its low decomposition temperature of about 400 °C that causes the complete volatilization of P, without any significant carbon residue. In addition, if any of these two components constituting the 3D (N)C residue somehow involved P as the precursor, their nitrogen content should probably increase from 3D (N)C-1 to 3D (N)C-4, as the percentage of P in the various P@CS-*n* precursors decreased in that order. However, this was not the case and the N content of the four 3D (N)C carbon residues under study was similar by about 6.4 wt %, which is the commonly found N content for carbon residues derived from CS.^24^ This suggests that both types of carbon residues present in the 3D (N)C-*n* samples must have come from CS. Scheme 2 depicts our proposal to justify the formation of the two elements observed by TEM in the carbon residue. This proposal is based on the ability of amphoteric P to self-assemble in nanometric droplets, resulting in the soft structuration of layered inorganic domains.^26,27^ According to this proposal, after the formation of P droplets, CS will form the micrometric sheets with some chains at the borders. These sheets will be increasingly curved due to the accumulated stress caused by the CS chains at the borders, causing the morphological changes toward hollow spheres, as previously indicated.

**Figure 4:**
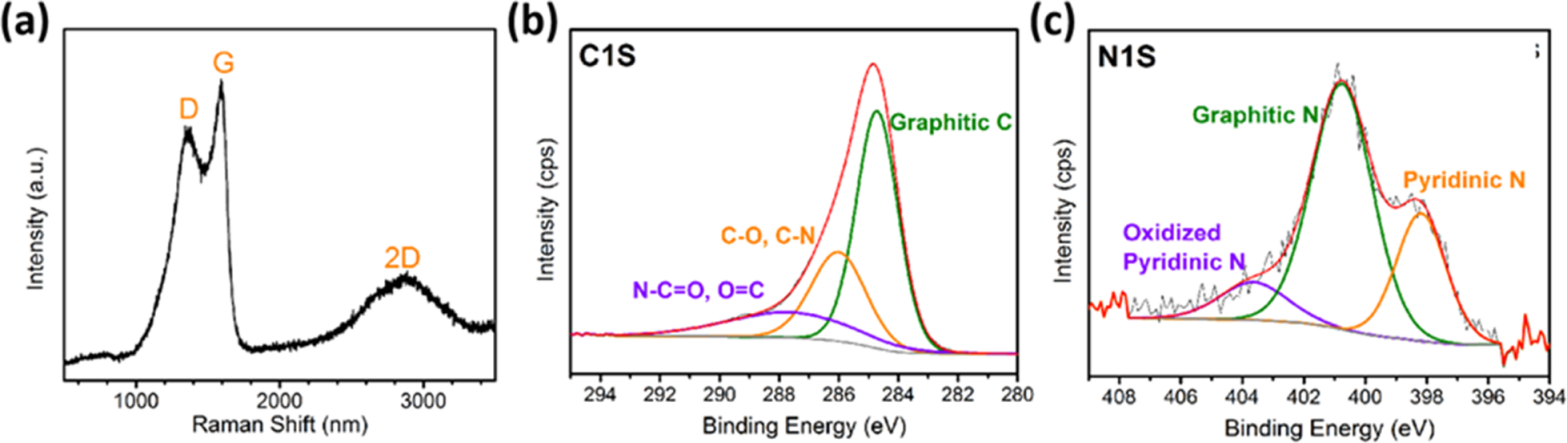
(a) Raman spectra; (b) high-resolution XPS C 1s spectrum; and (c) N 1s spectrum of 3D (N)C-2.

The transformation of CS into N-doped graphitic carbon after pyrolysis can be confirmed by Raman spectroscopy and X-ray photoelectron spectroscopy (XPS) analysis of the resulting 3D (N)C materials. In Raman spectroscopy, all 3D (N)G samples exhibit the characteristic 2D, G, and D peaks corresponding to defective N-doped graphene appearing at 2850, 1590, and 1350 cm^–1^, respectively. No shifts in the position of these three characteristic peaks or in the relative intensity of the G vs D bands were observed for the 3D (N)C samples under study. Moreover, they were also consistent with the Raman spectra of N-doped graphene obtained by the pyrolysis of CS in the absence of any P.^23^Figure 4a shows a representative Raman spectrum, while Figure S4 in the Supporting Information contains the spectrum of other samples.

Survey XPS analysis of the 3D (N)C samples shows the presence of C, N, and O in a similar atomic composition for all of the samples (Figure S5, Supporting Information). The high-resolution XPS C 1s peak for all of the samples was conveniently deconvoluted in the three individual components appearing at 284.5, 286.0, and 287.9 eV, attributable to graphitic C atoms, C atoms bonded to O or N with single or double bonds, and C atoms of carboxylic groups or amides, respectively. Figure 4b shows a representative XPS C 1s peak with the best fitting to its individual components, while Figure S5 in the Supporting Information contains the data for other 3D (N)C samples as well as the Table S1 in the Supporting Information lists the percentages of each type of carbon atom. As it can be seen there, all of the samples exhibit similar distribution of the different C atoms, suggesting that the proportion of P on the P@CS precursor of 3D (N)C-*n* does not play a role on the resulting graphitic material.

XPS analysis also revealed the presence of N in the four 3D (N)C in similar atomic percentage vs C of about 6.4%. N-doping was in agreement with the thermal behavior of CS that formed N-doped graphene. The high-resolution XPS N 1s peak was also appropriately deconvoluted into three main components, appearing at 398.2, 400.8, and 403.6 eV, attributable to pyridinic N, graphitic N, and oxidized pyridinic N, respectively. Figure 4c illustrates the distribution of the experimental XPS N 1s peak into the three major components of sample 3D (N)C-2 and Table S1 in the Supporting Information contains additional data of other samples. Again, different N families are in similar proportions for all of the samples. Of note is that the good fit between the experimental and deconvoluted N 1s peak (compare in Figure 4c the black and the red lines) indicates that pyrrolic N atoms should be present in the 3D (N)C samples in very low proportions.

**Figure 5:**
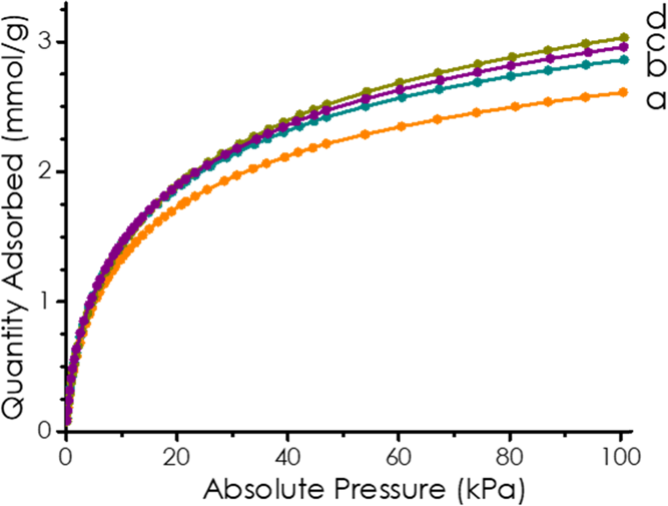
CO_2_ adsorption isotherms of (a) 3D (N)C-3; (b) 3D (N)C-1; (c) 3D (N)C-4; and (d) 3D (N)C-2 at 273 K.

Isothermal N_2_ adsorption at 77 K for these four 3D (N)C samples gave low specific area values below 100 m^2^ g^–1^. In contrast, these four samples exhibit a significant CO_2_ adsorption capacity. Figure 5 shows the plots of CO_2_ adsorption as a function of the pressure measured at 273 K. From these CO_2_ adsorption data, the specific surface area can also be determined. Table 1 summarizes the CO_2_ adsorption capacity as well as the specific area values at 100 kPa. These adsorption measurements show that these 3D (N)C carbon materials exhibit a significant specific surface area above 400 m^2^ g^–1^, reaching almost 500 m^2^ g^–1^. This discrepancy between N_2_ and CO_2_ adsorption data is probably due to a combination of factors, including the different temperatures of the adsorption measurement, the smaller kinetic diameter of CO_2_ (330 pm) compared to N_2_ (364 pm), and the different acid–base nature of the gases. Thus, in accordance with the literature,^28^ it is proposed that the combination of a large specific surface area, the presence of ultramicropores^29^ (pore diameter smaller than 0.5 nm), and the basicity due to pyridinic N atoms^30^ are the factors responsible for the high CO_2_ adsorption capacity of 3D (N)C materials. In any case, CO_2_ adsorption data show a significant porosity in the four 3D (N)C samples.

Regarding the CO_2_ adsorption capacity, sample 3D (N)C-2 exhibits the highest adsorption capacity among the series with a notable CO_2_ adsorption capacity of 3.03 mmol g^–1^ that is significantly higher than values reported in the literature for the related porous carbons. To put the value measured for 3D (N)C-2 into context, Table S2 in the Supporting Information summarizes other reported adsorption capacity values for CO_2_ of related porous carbon.^31−34^

### Photocatalytic Activity

In the context of the ongoing shift from fossil fuels to nonpolluting, renewable, and abundant energy, one strategy that is gaining importance is to convert solar energy into fuels, hydrogen being an attractive possibility.^35,36^ Most of the current photocatalytic systems under investigation are based on metals, but the use of metal-free materials is becoming increasingly appealing, particularly if they can be derived from biomass.^37^ Since it has been reported that N-doped graphene derived from CS is a visible-light photocatalyst for H_2_ generation in the presence of sacrificial electron donors,^38^ it would be of interest to assess if porous 3D (N)C materials also exhibit photocatalytic activity.

**Scheme 3:**
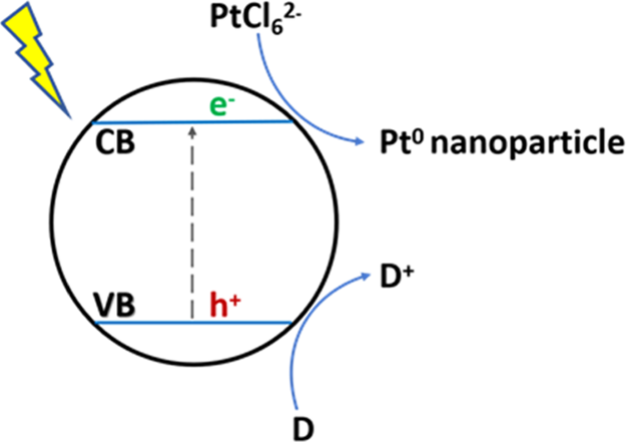
Schematic Diagram of Photodeposition of Pt Nanoparticles on a Photocatalyst by Photoinduced Electron Reduction D: Sacrificial electron donor, CB: conduction band, and VB: valence band.

One of the simplest, most-convincing evidence of the occurrence of photoinduced charge separation with the generation of conduction band electrons and valence band holes is the occurrence of metal photodeposition.^39^ In this experiment, an aqueous solution containing reducible PtCl_6_^2–^ in methanol as the sacrificial electron donor is subjected to irradiation in the presence of a photocatalyst, and the resulting solid is subsequently analyzed for the presence of Pt nanoparticles. The occurring reduction–oxidation reactions are indicated in Scheme 3.

Upon UV–vis irradiation of 3D (N)C-2 in an aqueous solution of K_2_PtCl_6_ containing 10 wt % CH_3_OH for 15 min, TEM images clearly reveal the formation of Pt nanoparticles that are characterized by measuring a lattice fringe of 0.224 nm that corresponds to the characteristic interplanar distance of the (111) planes in Pt (PDF#04-0802). Figure S6 in the Supporting Information provides illustrative images of the Pt nanoparticles formed by photodeposition. Control experiments under the same conditions in the dark do not show any Pt deposition.

**Figure 6:**
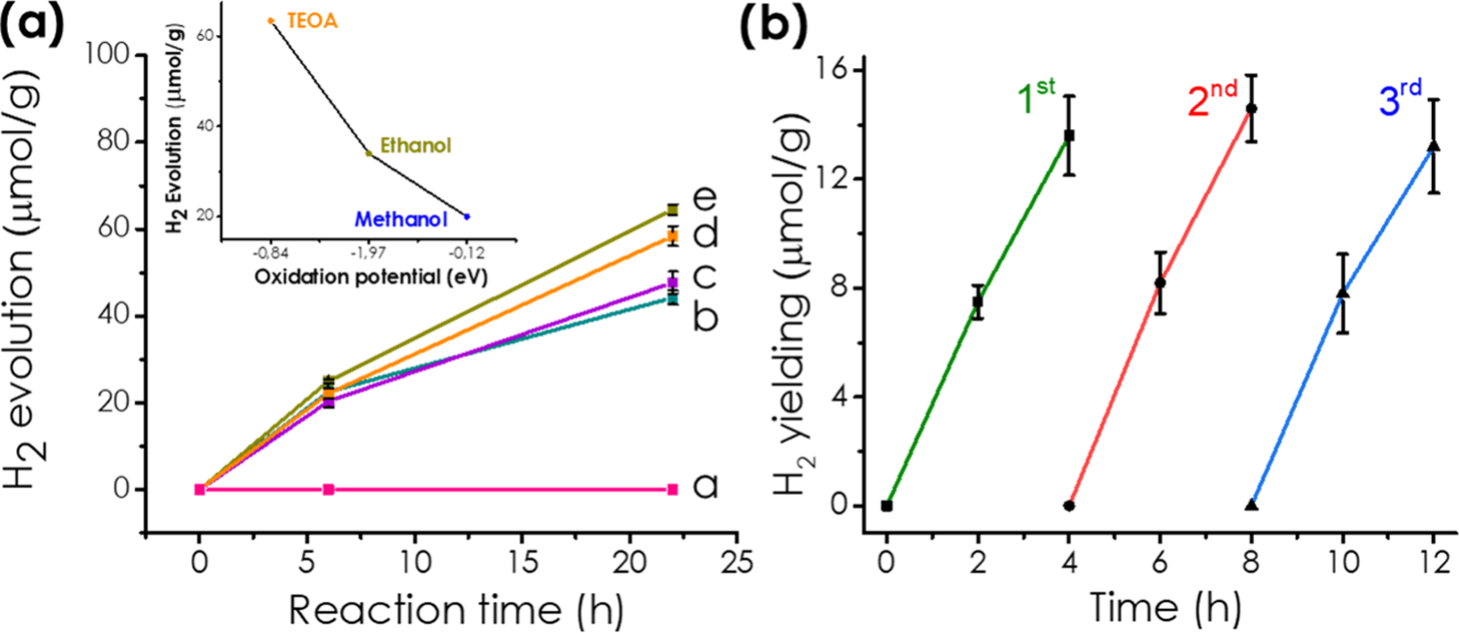
(a) Temporal profiles of hydrogen evolution using (a) blank, (b) 3D(N)C-1, (c) 3D(N)C-4, (d) 3D(N)C-3, and (e) 3D(N)C-2 as photocatalysts and TEOA as the electron donor. Inset: Plot of hydrogen evolution at 24 h using 3D (N)C-2 as the photocatalyst as a function of the oxidation potential of the sacrificial reagent (TEOA, ethanol, or methanol). (b) Three consecutive photocatalytic hydrogen evolution reactions with 3D(N)C-2 as the photocatalyst.

After having confirmed the photocatalytic charge generation by Pt photodeposition, the four 3D (N)C samples were screened as photocatalysts for H_2_ evolution in aqueous solution containing triethanolamine (TEOA) as the sacrificial electron donor. The results are presented in Figure 6a, showing that H_2_ evolves upon UV–vis irradiation of any of the four 3D (N)C, sample 3D (N)C-2 being the most active one. The stability of 3D (N)C-2 under photocatalytic conditions was assessed by performing three uses of the same sample, observing the same initial reaction rate and temporal profiles in the three consecutive runs (Figure 6b), thus confirming photocatalytic stability.

In agreement with Scheme 3 showing photoinduced charge separation, photocatalytic H^+^ or H_2_O reduction by conduction band electrons relies on the fast scavenging of conduction band holes by electron donors, as TEOA in the previous measurements. The ability of these sacrificial electron donors to quench holes depends on their oxidation potential, amines being better electron donors than alcohols and ethanol better than methanol. Thus, it has been frequently reported that H_2_ evolution depends on the redox potential of the electron donor, increasing as the electron donor oxidation potential decreases.^40^ This effect was observed here also, the H_2_ amount at a certain time increasing in the order of TEOA > ethanol > methanol (inset of Figure 6a).

As commented when describing the structure of 3D (N)C materials, these graphitic carbons are mainly constituted by micrometric 2D sheets accompanied by onion-like nanoparticles. To determine the intrinsic photocatalytic activity of the two components, the most active 3D (N)C-2 sample was subjected to ultrasounds to exfoliate the loose structure of these particles, allowing subsequently the suspension to sediment upon standing for a prolonged time. As previously discussed when commenting on TEM analysis shown in Figure 3, it was presumed that onion-like nanoparticles will remain mainly in the aqueous suspension, while large micrometric 2D sheets will slowly sediment. This assumption was confirmed by observation of the formation of a sedimented material, whose FESEM images correspond to the micrometric sheets, while FESEM images of the supernatant shows a considerable proportion of nanoparticles. Figure S7 in the Supporting information provides selected images of the two fractions obtained from 3D (N)C-2. Photocatalytic H_2_ generation in the presence of TEOA as the sacrificial electron donor shows that the two fractions exhibit activity with similar H_2_ evolution temporal profiles (Figure S8). After 24 h irradiation with simulated UV–vis light, the fraction corresponding to nanoparticles produced 64 μmol H_2_ × g^–1^, while the fraction mostly corresponding to the large micrometric sheets produced for the same time 50 μmol H_2_ × g^–1^. Thus, in spite of being the minor component, it seems that nanoparticles have higher intrinsic photocatalytic H_2_ activity than the predominant large sheet component. In any case, the combined photoresponse of the two fractions is larger than that of the pristine 3D (N)C particles, as presented in Figure 6a, of 64.4 μmol H_2_ × g^–1^ at 22 h, showing the benefits of particle dispersion and exfoliation.

**Figure 7:**
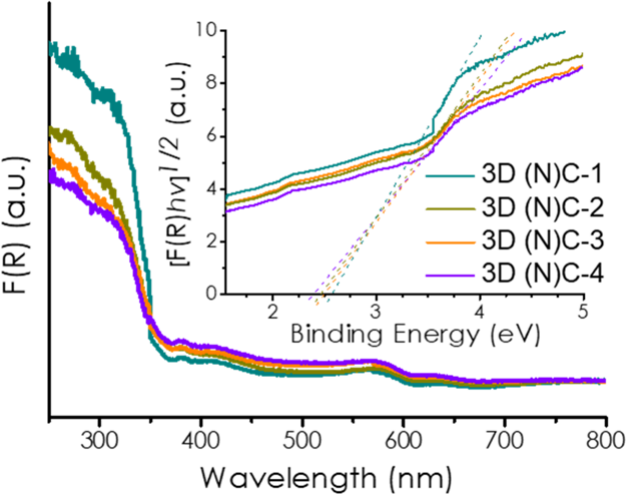
Diffuse reflectance UV–vis absorption spectra of 3D(N)C. Inset: Tauc plot and the estimated band gap values of the corresponding samples.

An estimation of the band gap energy was made based on the Tauc plot of the UV–vis absorption spectra. The four 3D (N)C samples present very similar absorption spectra with an intense peak in the UV region with an onset of about 380 nm and a series of weak absorption bands of decreasing intensity with relative maxima at 420, 560, and 640 nm (Figure 7). These weak bands are not present in N-doped graphene and it is proposed that they could be associated with the presence of carbon onion-like nanoparticles, which could explain its higher photocatalytic H_2_ generation activity. By extrapolation of the corresponding UV band in the Tauc plot, a band gap energy of 2.4–2.6 eV was estimated (inset plot of Figure 7).

At this point, it should be commented that none of the 3D (N)C materials was able to promote O_2_ evolution from H_2_O in the presence of Ce(IV) as the sacrificial electron acceptor agent. This failure is probably due to the insufficient oxidation potential of the valence band maximum in 3D (N)C-*n* that is dependent on the nature and density of the dopant elements, among other important factors. A similar failure to generate O_2_ was observed for N-doped graphene obtained by pyrolysis of CS that is a material related to the present tridimensional carbons.^38^ Also, in related precedents on the porous carbons, DFT calculations have indicated that unless there is a considerable density of oxygenated defects on the carbon composition, the energy of the valence band maximum is not sufficient to reach the oxidation potential required in the O_2_ evolution from H_2_O.^14^ These theoretical studies suggest that holes would be trapped on O atoms. Therefore, it seems that although doping of 3D (N)C opens a gap between the conduction and valence bands, the oxidation potential of the conduction band for these materials doped with N and O is not enough to promote H_2_O oxidation.

## Conclusions

The present article has shown that self-assembly of Pluronic P123 can produce structuration in the aqueous phase of a filmogenic natural polymer such as chitosan, resulting in the formation of particles of several micrometers with a defined morphology as orthoahedra or spheres with polar holes, as well as the intermediate morphologies depending on the Pluronic P123/chitosan mass ratio. These particles are constituted by the stacking of 2D sheets with a tendency to bend in the opposite concave–convex curvature in the upper and bottom faces, respectively. Upon pyrolysis, graphitic carbons are formed and the morphology is maintained, observing the expected micrometric continuous sheets corresponding to defective graphene, together with nanometric onion-like carbon particles with a graphitic structure. Although N_2_ adsorption in these porous carbon materials is very low, these N-doped carbons exhibit a significant CO_2_ adsorption capacity with a specific surface area of up to 499 m^2^ g^–1^, due to the combination of large surface areas, ultraporosity, and basic pyridinic N atoms. These N-doped carbons exhibit photocatalytic activity for H_2_ generation upon irradiation with UV–vis light in the presence of sacrificial electron donors, reaching a H_2_ production value of 64.4 μmol g^–1^ in 22 h. This photocatalytic activity is derived from both onion-like carbon nanoparticles and micrometric sheets, although nanoparticles appear to be somewhat more active. The present results illustrate the still poorly explored vast potential of structuration of organic compounds and polymers to obtain a variety of porous carbons with a defined particle morphology.

## Experimental Section

### Sample Preparation

Commercially available reagents were purchased from Aldrich and used without further purification. In a general preparation, 234 mg of Pluronic P123 from Aldrich and 1.18 mL of 37 wt % hydrochloric acid were dissolved in 7.56 mL of Milli-Q H_2_O at 50 °C. In a different flask, 325 mg of chitosan and 203 μL of acetic acid were dissolved in 16.25 mL of Milli-Q H_2_O. After 4 h of magnetic stirring to ensure complete dissolution, the chitosan aqueous solution was added into the Pluronic P123 aqueous solution under magnetic stirring. The mixed solution was further stirred for 6 h at room temperature to ensure complete homogeneity. Then, the solution was transferred to a Teflon-lined autoclave and heated at 100 °C under autogenous pressure for 24 h. This hydrothermal treatment produces templation of chitosan fibrils by Pluronic P123 micelles. The P@CS samples were obtained by water evaporation at 50 °C. Different materials were similarly prepared varying Pluronic P123 and chitosan weights as indicated in Table 1. To obtain 3D@(N)C, the P@CS samples were pyrolyzed under an Ar flow (200 mL min^–1^), increasing the temperature at a rate of 2 °C min^–1^ up to 900 °C and holding for 2 h. After this time, the oven was allowed to cool down at room temperature maintaining the Ar flow.

### Characterization

FESEM images were acquired with a JEOL JSM 6300 apparatus. HRTEM images were recorded in a JEOL JEM 2100F under an accelerating voltage of 200 kV. Samples were prepared by applying one drop of the suspended material by tip sonication (700 W) for at least 15 min in ethanol onto a carbon-coated nickel TEM grid and allowing it to dry at room temperature. Raman spectra were collected with a Horiba Jobin Yvon-LabRAM HR UV–visible–NIR (200–1600 nm) Raman microscope spectrometer using a 512 nm laser for excitation. The spectra were collected by averaging 10 scans at a resolution of 2 cm^–1^. XPS was measured on a SPECS spectrometer equipped with a Phoibos 150 9MCD detector using a nonmonochromatic X-ray source (Al and Mg) operating at 200 W. The samples were evacuated in the prechamber of the spectrometer at 1 × 10^–9^ mbar. The measured intensity ratios of the components were obtained from the area of the corresponding peaks after nonlinear Shirley-type background subtraction and corrected by the transition function of the spectrometer. The chemical composition of the samples was determined by combustion chemical analysis using a CHNS Fisons elemental analyzer. The micropore volume and specific surface area of the solids were measured by N_2_ adsorption isotherms at −196 °C, using a Micromeritics ASAP 2010 instrument. The CO_2_ adsorption isotherms in the low-pressure range were measured using a Micromeritics ASAP 2010 instrument using approximately 200 mg of the solid placed in a sample holder, which was immersed into a liquid circulation thermostatic bath for precise temperature control. Before each measurement, the sample was treated overnight at 400 °C under vacuum. CO_2_ adsorption isotherms were then acquired at 0, 10, 20, 30, and 40 °C.

### Photocatalytic Tests

All of the photocatalytic tests were performed using a 51 mL cylindrical quartz photoreactor fitted with a manometer, an inlet, and an outlet valve under irradiation with UV–vis light from a 300 W Xe lamp. Typically, 20 mg of the catalyst was dispersed in 20 mL of aqueous solution with a 10 vol % electron donor. The system was then purged with Ar for 15 min before irradiation. The evolved H_2_ was analyzed using a gas chromatograph (Agilent 490 MicroGC) equipped with a molecular sieve 5 Å column with a TC detector and Ar as the carrier gas. A photocatalytic H_2_ evolution test was repeated five times to determine the average value and standard deviation.

To determine the relative activity of onion-like carbon nanoparticles with respect to the micrometric 2D sheets, 10 mg of 3D (N)C was dispersed in 10 mL of Milli-Q water using the tip of an ultrasound generator OF 700 W operating in pulse mode with 1 s pulses on and a resting time of 10 s during 2 h. After this time, the suspension was allowed to sediment for 5 h. Then, the supernatant was decanted and used directly in photocatalytic H_2_ generation under the general conditions indicated above. The bottom solid was redispersed in 9 mL of Milli-Q water and 1 mL of TEOA and the suspension was also subjected to irradiation to evaluate the H_2_ generation activity.

### Photodeposition of Pt

20 mg of the catalyst was dispersed in 18 mL of the 1 mM H_2_PtCl_6_ solution by sonication, and then 2 mL of methanol was added to the dispersion as the electron donor. The aqueous solution was purged with Ar to evacuate O_2_ from the system before irradiation. After 15 min irradiation with a Xe lamp, the black solid was collected by centrifugation. Then, the obtained catalyst was exhaustively washed with Milli-Q water and characterized by HRTEM and DFTEM. A control experiment in the dark was performed following an identical procedure but completely wrapping the 51 mL photoreactor with Al foil to avoid light into the suspension.

## Supporting Information Available

FESEM images of P@CS-*n*; additional FESEM and TEM images of 3D (N)C; Raman and XP spectra of 3D of (N)C; high-angle annular dark-field and TEM images of Pt nanoparticles on 3D (N)C-2; FESEM images and photocatalytic activity of the supernatant and sediment upon dispersion of 3D (N)C-2; and components of XPS peak deconvolutions and comparison of CO_2_ adsorption capacity of 3D (N)C with other carbonaceous materials in the literature (PDF)
