# Supplementary material for: Porous Graphitic Carbons Containing Nitrogen by Structuration of Chitosan with Pluronic P123
Source: ACS Appl Mater Interfaces. 2021 Mar 11;13(11):13499–507. doi: 10.1021/acsami.0c19463 (PMC8528379; doi:10.1021/acsami.0c19463)
Supplement: Supplementary file 1 — am0c19463_si_001.pdf [file am0c19463_si_001.pdf]

Supporting Information

Porous Graphitic Carbons Containing Nitrogen by Structuration of Chitosan with Pluronic

Lu Peng, Yong Peng, Ana Primo and Hermenegildo García**

Instituto Universitario de Tecnología Química, Universitat Politècnica de València-Consejo Superior de Investigaciones Científicas, Av. de los Naranjos s/n, 46022 Valencia, Spain. E-mail addresses: aprimoar@itq.upv.es and hgarcia@qimn.upv.es

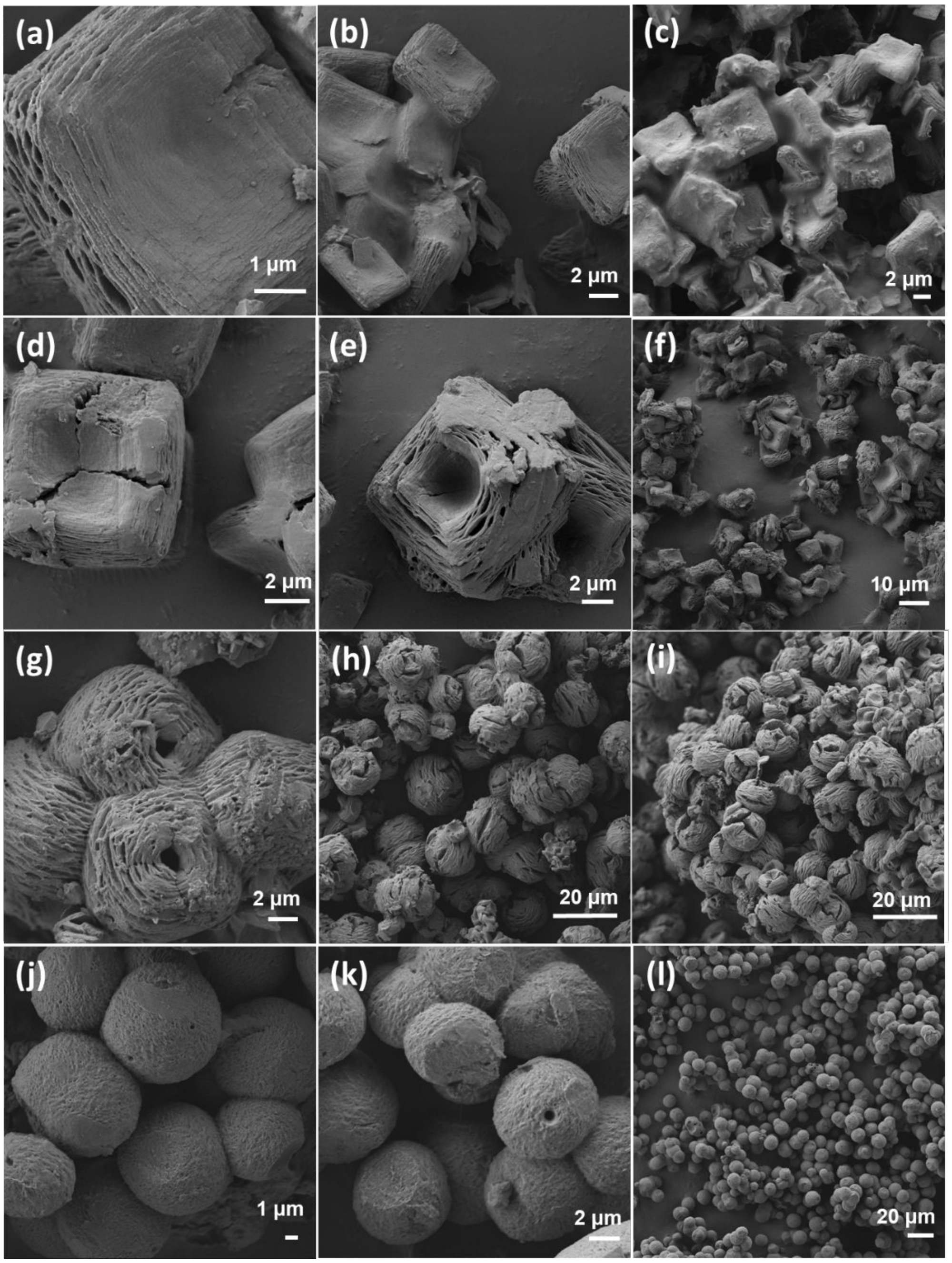

Figure S.1. . FESEM images of a, b, c) P@CS-1; d, e, f) P@CS-2; g, h, i) P@CS-3 and j, k, l) P@CS-4.

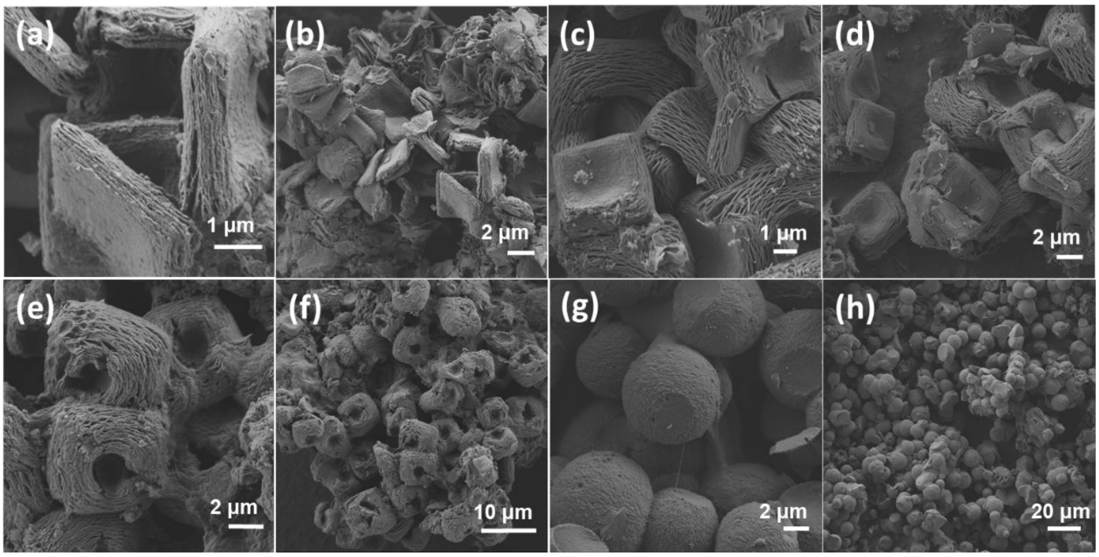

Figure S.2. FESEM images of a,b) 3D (N)C-1; c, d) 3D (N)C-2 e, f) 3D (N)C-3 and g, h) 3D (N)C-4.

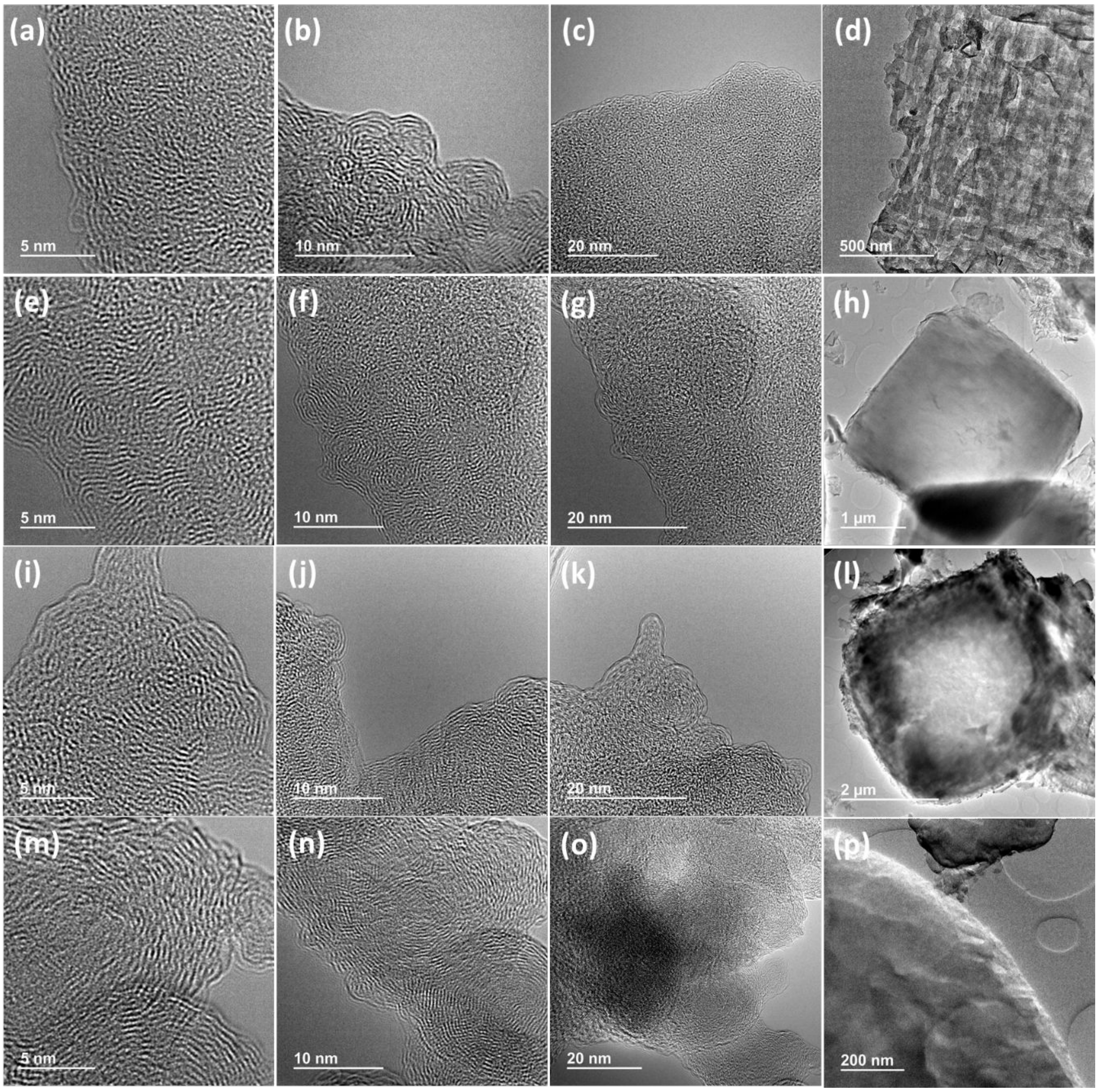

Figure S.3. TEM images of a, b, c, d) 3D (N)C-1; e, f, g, h) 3D (N)C-2; i, j, k, l) 3D (N)C-3 and m, n, o, p) 3D (N)C-4.

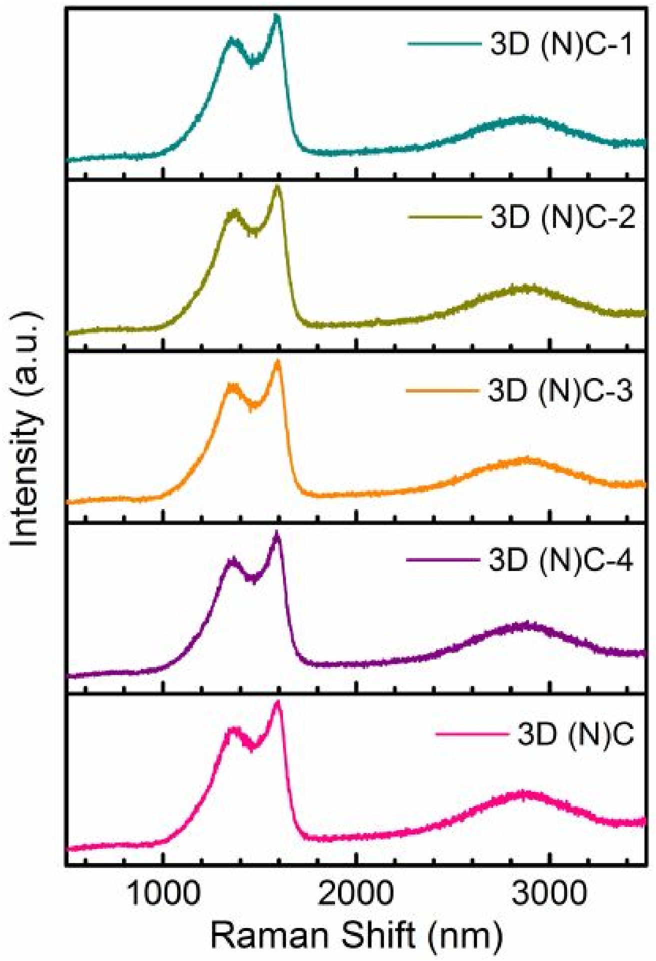

Figure S.4. Raman spectrum of 3D (N)C-n. 3D (N)C was prepared without Pluronic P123.

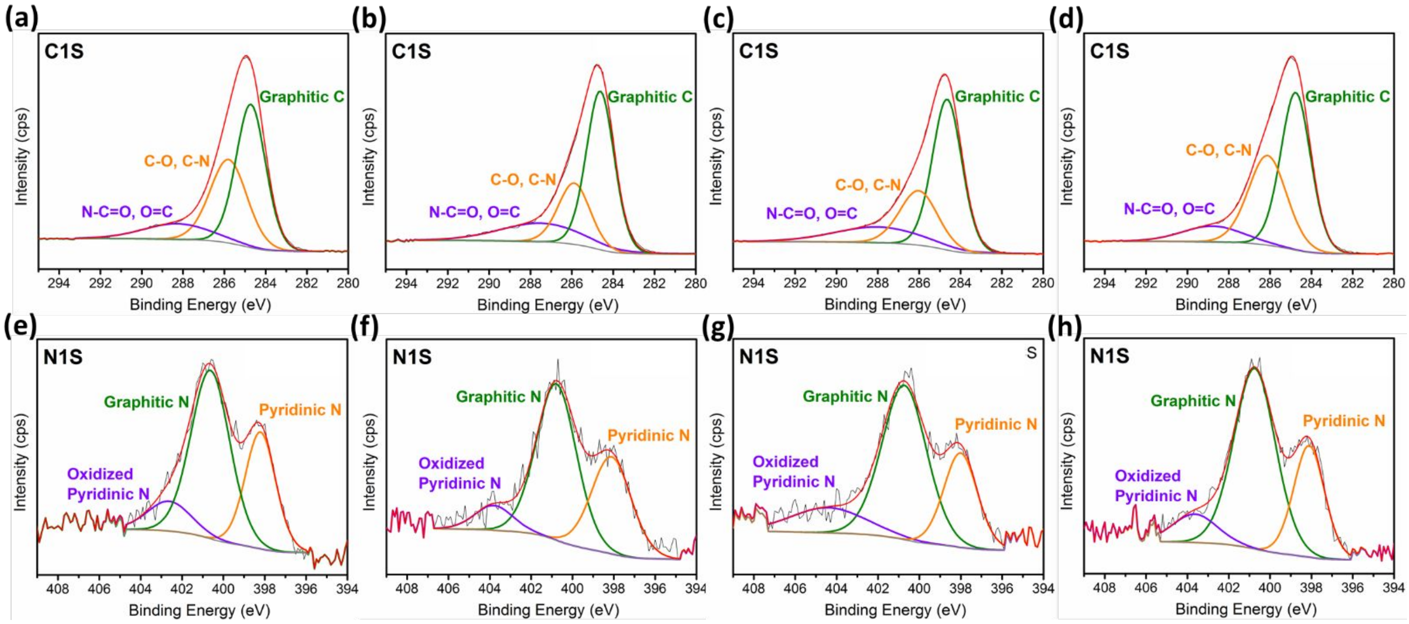

Figure S.5. High-resolution XPS C1s spectrum and N1s spectrum of a), e) 3D (N)C-1; b, f) 3D (N)C-3; c, g) 3D (N)C-4 and d, h) 3D (N)C.

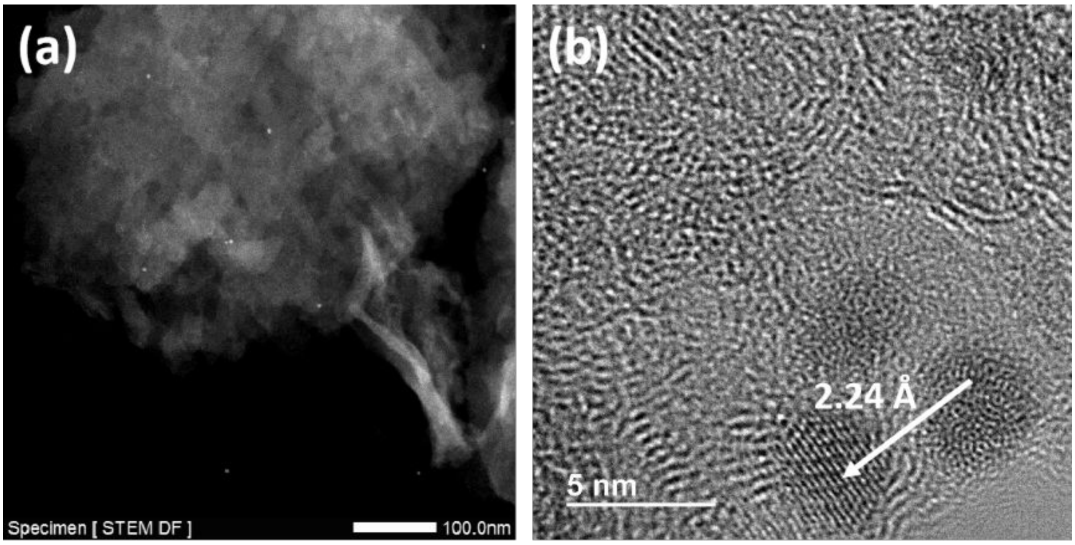

Figure S.6. a) High-angle annular dark-field (HAADF) images taken in STEM mode and b) HRTEM images taken in transmission mode of 3D (N)C-2 after irradiation in the presence of H_2PtCl_6, where the formation of small Pt nanoparticles can be clearly observed after irradiation.

A

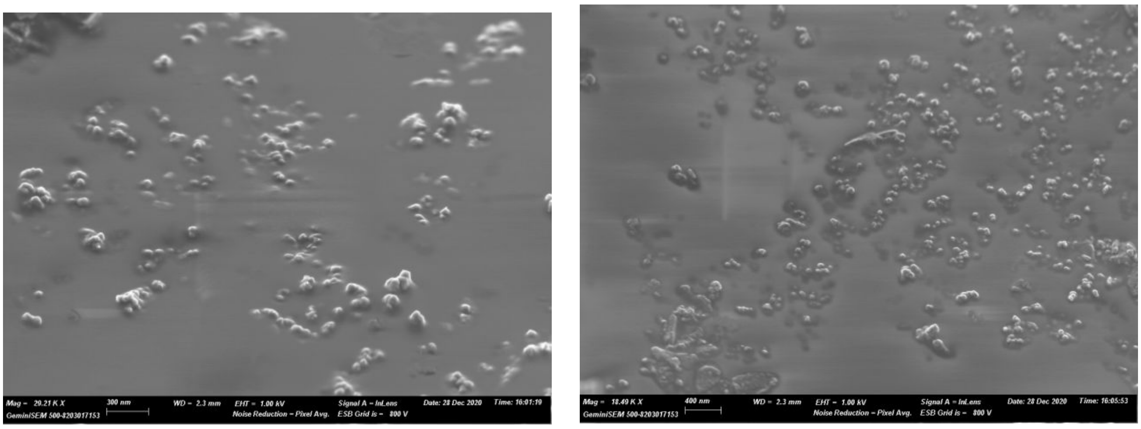

B

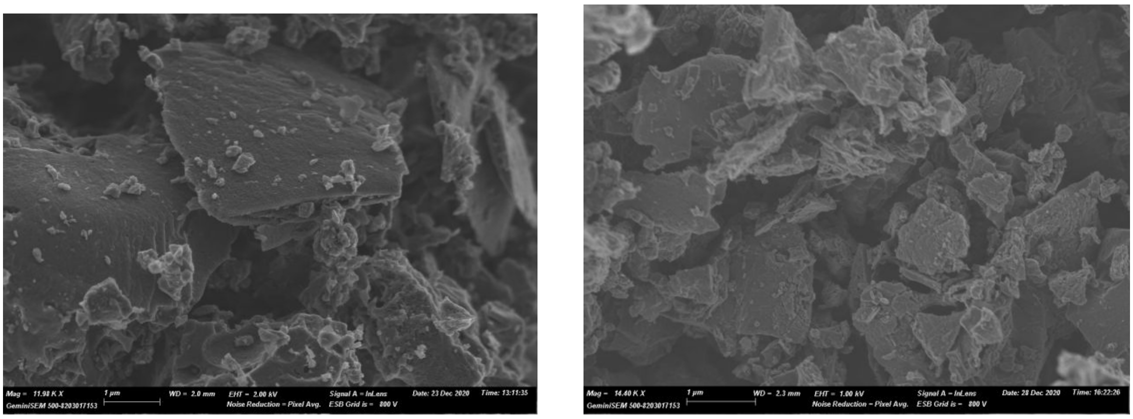

Figure S7. FESEM images of the material present in the supernatant (A) and the sediment (B) after sonication of 3D (N)C-2 and allowing the dispersion to sediment for 5 h.

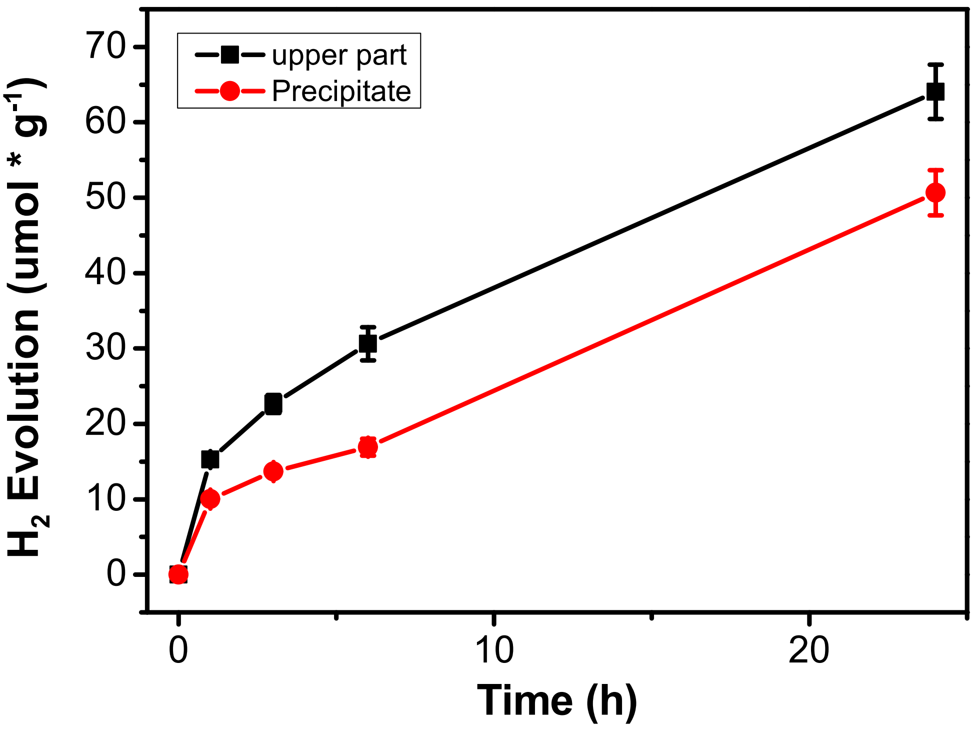

Figure S8. Photocatalytic activity of the upper and bottom material after dispersing 3D (N)C (10 mg) in H_2O (10 ml) ultrasounds and allowing to sediment the larger particles for 5 h. Irradiation conditions: 300 W Xe lamp, 10 vol.% TEAO as sacrificial electron donor, volume 10 mL, ambient temperature.

Table S.1. List of different types of carbon and nitrogen family based on high resolution. experimental XPS peaks.

Sample	Graphitic C [%]	C-O, C-N D-[%]	N-C=O, O=C [%]	Graphitic N [%]	Pyridinic N [%]	Oxisized Pyrindinc N [%]
3D (N)C-1	50.6	36.7	12.6	57.3	31.3	11.4
3D (N)C-2	55.2	27.0	17.8	60.7	26.6	10.7
3D (N)C-3	55.9	24.7	19.4	56.5	34.6	8.6
3D (N)C-4	57.8	26.3	17.4	58.7	26.5	15.9
3D (N)C	56.2	34.4	9.6	61.5	26.3	10.3

Table S.2. CO_2 capture comparison of different porous carbon materials.

Sample	S [m^2 g^{-1}]	Q_{max} [mmol g^{-1}]	Ref.
3D (N)C-1	477	2.96	This work
3D (N)C-2	499	3.03	This work
3D (N)C-3	402	2.61	This work
3D (N)C-4	441	2.86	This work
GO	4.27	0.166	[1]
GO-EDA-0.2	18.2	1.461	[1]
T-G-700-6	1296	2.52	[2]
GU-700-6	360	1.43	[2]
P-g-C_3N_4	13.4	0.39	[3]
g-C_3N_4	3.0	0.12	[3]
CDMC	871	1.31	[4]

Supplementary References

1. An, L.; Liu, S.; Wang, L.; Wu, J.; Wu, Z.; Ma, C.; Yu, Q.; Hu, X., Novel Nitrogen-Doped Porous Carbons Derived from Graphene for Effective CO2 Capture, Ind. Eng. Chem. Res. 2019, 58, 3349-3358.

2. Cai, J.; Chen, J.; Zeng, P.; Pang, Z.; Kong, X., Molecular Mechanisms of CO2 Adsorption in Diamine-Cross-Linked Graphene Oxide, Chem. Mater. 2019, 31, 3729-3735.

3. Liu, B.; Ye, L.; Wang, R.; Yang, J.; Zhang, Y.; Guan, R.; Tian, L.; Chen, X., Phosphorus-Doped Graphitic Carbon Nitride Nanotubes with Amino-Rich Surface for Efficient CO2 Capture, Enhanced Photocatalytic Activity, and Product Selectivity, ACS Appl. Mater. Interf. 2018, 10, 4001-4009.

4. Peng, H.-L.; Zhang, J.-B.; Zhang, J.-Y.; Zhong, F.-Y.; Wu, P.-K.; Huang, K.; Fan, J.-P.; Liu, F., Chitosan-Derived Mesoporous Carbon with Ultrahigh Pore Volume for Amine Impregnation and Highly Efficient CO2 Capture, Chem. Eng. J. 2019, 359, 1159-1165.
